# Temporal processes in prime–mask interaction: Assessing perceptual consequences of masked information

**DOI:** 10.2478/v10053-008-0028-x

**Published:** 2008-07-15

**Authors:** Ingrid Scharlau

**Affiliations:** Department of Psychology, Bielefeld University, Germany

**Keywords:** masked priming, attention, visual backward masking, metacontrast, perceptual latency, perception of time

## Abstract

Visual backward masking is frequently used to study the temporal dynamics of visual perception. These dynamics may include the temporal features of conscious percepts, as suggested, for instance, by the asynchronous–updating model (Neumann, 1982) and perceptual–retouch theory ((Bachmann, 1994). These models predict that the perceptual latency of a visual backward mask is shorter than that of a like reference stimulus that was not preceded by a masked stimulus. The prediction has been confirmed by studies using temporal–order judgments: For certain asynchronies between mask and reference stimulus, temporal–order reversals are quite frequent (e.g. Scharlau, & Neumann, 2003a). However, it may be argued that these reversals were due to a response bias in favour of the mask rather than true temporal-perceptual effects. I introduce two measures for assessing latency effects that (1) are not prone to such a response bias, (2) allow to quantify the latency gain, and (3) extend the perceptual evidence from order reversals to duration/interval perception, that is, demonstrate that the perceived interval between a mask and a reference stimulus may be shortened as well as prolonged by the presence of a masked stimulus. Consequences for theories of visual masking such as asynchronous–updating, perceptual–retouch, and reentrant models are discussed.

## INTRODUCTION

Visual masking has, for a considerable amount of time, proven to be a powerful tool for investigating the temporal dynamics of visual perception. One prominent method within this research tradition is to demonstrate that although there is no conscious perception of a masked stimulus (a ‘prime’), the features or presence of the masked information may influence sensorimotor (e.g., Klotz & Neumann, 1999), attentional (e.g., Jaśkowski, van der Lubbe, Schlotterbeck, & Verleger, 2002), semantic (e.g., Kiefer, 2002, this volume), and mental operations (e.g., Mattler, 2003). However, as pointed out, among others, by Schmidt and Vorberg (2006; Schmidt, this volume), the requirement that awareness of the masked information is perfectly absent is both difficult to prove and to achieve. Schmidt and Vorberg advocate a technique in which one attempts to demonstrate that an independent variable influences awareness and other measures of processing differently, instead of trying to prove that a prime is completely masked.

Yet, non–chance perception of the prime, or ‘residual awareness’, is more than a *problem* for masking research. Whether a masked stimulus leaves traces in perception – and how to assess them – is a *research question in its own right*. For example, features of a masked stimulus may migrate to the mask (feature inheritance), and the spatio-temporal conditions under which total masking, feature inheritance, or other phenomena dominate allows insights into the time course of visual information processing (e.g., Herzog, Fahle, & Koch, 2001; Herzog, Koch, & Fahle, 2001).

In the present paper, I propose to study whether the masked prime influences temporal perception. Previous studies have indicated that priming alters *temporal features* of the *consciously perceived mask*: Given a pair of a masking stimulus and a reference stimulus that is not preceded by a prime, the mask appears to begin earlier. That is, if observers, for instance, report which of two simultaneous stimuli – mask and reference – is the earlier one, they will tend to choose the mask, not the reference (perceptual latency priming or PLP; Scharlau & Neumann, 2003a).

Within the framework of masking research, PLP is not an accidental finding. It had been predicted by two models which aimed at explaining metacontrast masking, perceptual retouch (Bachmann, 1984) and the asynchronous–updating model (Neumann, 1982). Both ascribe metacontrast (and PLP) to the interaction and asynchrony of two processing mechanisms, one specific, the other more general. They differ, however, with respect to pinpointing these mechanisms.

According to the *asynchronous-updating model* (AUM), the onset of a stimulus causes two parallel visual processes: feature/object coding of basic visual information in spatially addressable maps on the one hand, and allocation of attention on the other hand. Whereas the first process is fast and reflects stimulus changes quickly, the second process is slow, lagging behind the information that is represented in the feature maps. Yet, it is a necessary precondition for conscious perception. To put it very generally, during the shift of attention towards the prime, the prime’s codes on the level of spatial maps are overwritten by the mask’s codes and thus prevented from attention-related processing. This model is able to explain why metacontrast masking can be reduced if attention is pre–cued towards the location of the prime–mask sequence (Enns, 2004; Tata, 2002) or when primes are attention-grabbing stimuli (Shelley-Tremblay & Mack, 1999), and conversely is increased if a distractor diverts attention away from the prime for longer prime-mask intervals (50 to 100 ms; Neumann, 1978).

According to the AUM, the most characteristic pattern of metacontrast masking, the U–shaped masking curve, is due to two separate mechanisms. Within the descending branch where masking increases with the temporal interval between prime and mask, temporal integration of prime and mask dominates, that is, they are perceived as a single event. Masking here is due to factors such as brightness summation. In the ascending branch, with masking decreasing, temporal differentiation dominates. Here, masking is related to attentional factors (Neumann, 1978). Reeves (1982, 1986) has provided further evidence for this decomposition of the masking curve into two mechanisms, and the idea has recently reappeared in masking research (von Mühlenen, Enns, & Di Lollo, 2006).

In the AUM, PLP is a by–product of the attention shift triggered by the prime: Attention-related processing of the mask can begin earlier, because the attention shift towards the location of the prime–mask sequence has already begun or been completed. This earlier beginning results in perceptual latency priming (Neumann, 1982).

In the *perceptual–retouch model*, the two asynchronous processes are fast specific encoding of information in the visual cortex (features, conjunctions/objects and intermodal binding), and slow nonspecific activation of these codes via retino-thalamic and thalamo-cortical pathways, which modulates specific afferent processes and is necessary for conscious availability of contents (see, e.g., Baars, 1995). Nonspecific processing or perceptual retouch modulates the specific codes in such a way that they are upgraded into conscious experience (Bachmann, 1994). Because nonspecific activation trails specific processes by about 50 to 80 ms (Bachmann, 1994), upon its arrival, the codes of the prime and the mask will vary – depending on priming/masking SOA (stimulus onset asynchrony) – in their strength, which in turn determines whether they will be upgraded or not. With short priming SOAs, prime and mask are upgraded as an integrated percept because both are similarly strong. With medium priming SOAs around 50 ms, the mask’s codes are strong enough for upgrading while those of the prime have already decayed, and with large priming SOAs, both stimuli are upgraded separately. This explains the U-shaped function of metacontrast by a single mechanism.

PLP is included in the perceptual-retouch model via the beginning of the upgrading or retouch process: Because this process begins earlier for a primed mask compared to an unprimed stimulus, the mask’s perceived onset is pre–dated. Processing of the mask on the level of upgrading takes advantage of the nonspecific activation triggered by the prime (Bachmann, 1999).

As mentioned above, latency facilitation of the mask, which was expected on the basis of these two models, has indeed been found in several studies which compared the perceptual latency of the mask and a reference stimulus in temporal–order judgments (Neumann, 1982; Neumann, Esselmann, & Klotz, 1993; Scharlau & Neumann, 2003a; Steglich & Neumann, 2000). Several features of PLP, such as its time course (Scharlau, Ansorge, & Horstmann, 2006), its independence of sensorimotor processing (Scharlau, 2004), its independence of prime-target similarity (Scharlau & Neumann, 2003a), and the possibility of top-down influences (Scharlau & Ansorge, 2003) accord well with the attention-related explanation of the asynchronous–updating model (for a summary of the empirical data see Scharlau, in press). However, it is still not clear whether mechanisms such as decision–level processing or the establishment of judgment criteria contribute to PLP. This is mainly due to a shortcoming of the usual method of measuring latency facilitation, temporal–order judgment (TOJ).

TOJ is a very natural method to assess PLP: With its help, the latency of the primed stimulus is compared directly to the latency of an unprimed stimulus. TOJ data allow quantifying the latency gain and measure discrimination accuracy simultaneously (e.g., Sternberg & Knoll, 1973). A disadvantage of the TOJ method is that it does not provide easy means to distinguish between ‘true’ latency effects and criterion effects (for further shortcomings, see Ulrich, 1987).

Several authors have argued that evidence in favour of PLP (or similar attentional effects) may alternatively be caused by a non-attentional change in response or decision criteria, that is, a *response bias* (Jaśkowski, 1993; Pashler, 1998). In the following, I will shortly explain the response–bias argument, describe how it has been addressed in earlier research and point out the shortcomings of these earlier attempts. Then I will propose two related methods to assess PLP that are less prone to response bias. The three tasks are then studied jointly in two experiments.

### The response–bias argument

In general, the response-bias account of PLP argues that if in doubt, observers may tend to ascribe a response or judgment criterion – in the TOJ, the criterion “being the first stimulus” – to the primed stimulus or the mask. This objection was first raised by Jaśkowski (1993), although restricted to conditions in which the actual interval between the stimuli was so short that order was difficult to perceive and the observer was uncertain about it. However, one may, as Pashler did (1998) in a review of cueing research, generalise such doubts: Observers may in general tend to respond in favour of an attended or primed stimulus.

Shore, Spence, and Klein (2001) studied this in a temporal–order judgment task with attentional cueing. They compared latency facilitation^1^ in judgments with opposite temporal criteria (“first” and “second” judgments) and defined true latency facilitation as the mean of these two conditions and response bias as half the difference between the two conditions. With endogenous cueing by centrally presented arrows they found that the response bias was approximately as large as the latency facilitation itself (13 vs. 17 ms). With exogenous cueing, the same response bias of 13 ms was present, but small compared with a large latency benefit (61 ms). In a similar study, although with masked primes, I found no response bias (Scharlau, 2004).

Thus, the question of response bias in latency facilitation is still unsettled. First, masked primes may not elicit a bias. Further, the study of Shore et al. (2001) might be in need of replication because there were only three observers per condition, and the *PSS* (the point of subjective simultaneity) was calculated from only two data points on the psychometric distribution, a procedure falling short of psychophysical methods which estimate the parameters from the whole distribution (Finney, 1971; Thurstone, 1948).

In the present study, I attempt to test methods to measure PLP which narrow possible influences of a response bias. As explained, a response bias may interfere in PLP experiments because observers give a two-alternative forced-choice judgment, and attention is primed to either one of two alternative features (locations or stimuli). A dependent measure that consists of more than two alternative responses precludes such a response bias because it prevents a criterion from being ascribed to the primed stimulus.

There are different possibilities for realising such a method. Shore et al. (2001) proposed using *judgment times*. They reasoned that the most difficult order judgments should yield the longest judgment times. The peak of judgment times thus indicates perceived simultaneity. Indeed they found that judgment times peaked approximately at the temporal intervals that defined the *PSS*. Further, the peaks were shifted in accordance with latency facilitation, that is, the direction of the peak shift was the same as that of the *PSS* shift. The method has, however, disadvantages: In the judgment–peak analysis, a single data point (peak judgment time) is taken as the index of *PSS*. This method falls short of psychophysical threshold analysis which is used to estimate the *PSS* from temporal order judgments (see, e.g., Finney, 1971): The precision with which the peak is determined depends on an appropriate choice of temporal intervals that are used as the independent variable. Furthermore, only the peak is extracted from the data. By comparison, threshold analysis yields a measure of discrimination performance (difference limen, *DL*) besides *PSS*. Most importantly, if the distributions do not peak sharply it is arguable whether any single point on this distribution marks the *PSS*. This is evident in the study by Shore et al.: Although there was clear evidence for latency facilitation in the psychometric distributions and *PSS* data, the peaks in judgment times were poorly localized because of shallow slopes.

One further disadvantage of peak analysis may be added. Judgment times are often highly variable, and this variability renders statistical evaluation difficult. Further, either correct and incorrect judgments or correct judgments only can be tested. PLP is by definition accompanied by a change of error rate which speaks in favour of using both correct and incorrect judgments. However, errors may result from multiple causes besides latency facilitation (cf. Reason, 1990). Including them will thus further increase variance and complicate statistical testing.

### Alternative methods

In the following, I describe two alternative methods of measuring latency facilitation that avoid the shortcomings discussed in the previous paragraphs. They are meant to prevent a response bias, allow for computing parameters of temporal perception which are comparable to the parameters of psychophysical threshold analysis and permit statistical treatment, and extend the evidence of PLP to the perception of duration. Two experiments each compare three tasks: *temporal order judgment* (TOJ), *interval reproduction*, and *interval scaling*.

The TOJ serves as a comparison for the two novel methods. It was used in all of the earlier studies on PLP. Observers judge which of two visual targets appears first. In order to test the proposal by Shore et al. (2001), I will also analyse the judgment times.

In the reproduction and scaling parts – the two new methods –, observers judge the *duration of the perceived interval* between the two targets’ onsets by manual reproduction and by a graphic scale, respectively. Latency facilitation by the prime should lead to a prolongation of the perceived interval if the prime precedes the first target because here, PLP speeds up the processing of the target that marks the beginning of the interval. Conversely, intervals will be shortened if the prime precedes the second target because attention now speeds up the target that defines the end of the interval.

The TOJ task is a classical psychophysical method for estimating the thresholds of temporal perception. The scaling task may be conceived of as a method of direct scaling (cf. Stevens, 1957); the estimate is, however, given graphically and not numerically. The reproduction task is a motor task whose requirements are different both from judgment and direct scaling and which falls outside the scope of psychophysics. These three tasks are sufficiently dissimilar to underline the generality of PLP – provided that they yield converging evidence for PLP.

The two experiments use two different priming intervals (the interval between the onset of the prime and the onset of the mask), 37.5 and 67.5 ms. Both the AUM and the perceptual–retouch theory predict that PLP increases with priming interval within this range (Scharlau et al., 2006).

## EXPERIMENTS 1A AND 1B

### Method

#### Participants

Ten student participants gave their informed consent in Experiment 1a, and 11 participants in Experiment 1b. Each received € 15. The most accurate participant in each block gained an additional payment of € 3. All participants had normal or fully corrected vision.

#### General Design and Apparatus

Each experiment consisted of three blocks (TOJ, reproduction and scaling) run in random order on separate days. Dark grey stimuli were presented on a light grey background on a 17 in. colour monitor. Participants sat in a dimly lit room, with their line of gaze straight ahead and viewing distance fixed at 60 cm by a chin rest. They responded via a serial mouse.

#### Stimuli

In each trial, two targets were displayed, a square and a diamond. The pair appeared in horizontal alignment. It was always presented in the upper part of the screen, because in the scaling task, the response device was presented in the lower part, and I wanted to prevent any interaction of this with the relevant targets. Edge length of the targets was 2.3° of visual angle. Eccentricity was 8.5°, that is, the target centre was 6° from the centre of the screen both in horizontal and vertical direction. In half of the trials, a smaller version of a target (a prime) preceded one of the targets; edge length of the prime was 1.7°. The prime was visually backward-masked by the target at the same location (Klotz & Neumann, 1999).

The targets were presented with temporal intervals of –97.5 to +97.5 ms in steps of 15 ms (target SOAs, stimulus onset asynchronies). Negative numbers indicate that the primed target (or primed mask) preceded the unprimed target, and positive numbers denote that the unprimed target appeared first. (In trials without a prime, positive and negative numbers were assigned randomly while realising all the other variables equally often.) The priming SOA was 67.5 ms in Experiment 1a and 37.5 ms in Experiment 1b. All stimuli had durations of 37.5 ms. There were 28 conditions (2 priming conditions × 14 target SOAs; 672 trials). The trials were presented with the method of constant stimuli.

#### Procedure

Participants fixated on the centre of the screen, marked by a cross, throughout each trial. In the TOJ part, they indicated which of the two shapes was perceived first. Half of the participants pressed the right button of a computer mouse if the diamond appeared first, and the left button if the square was perceived first. For the other half, this assignment was reversed. The instruction emphasised accuracy.

In the reproduction part, participants reproduced the perceived interval with the mouse buttons. They pressed the two mouse buttons corresponding to the succession of the two targets as exactly as possible. They used their two index fingers for the reproduction, beginning with the left index finger if the left target led the sequence, and with the right index finger if the right target was first. The time between the two click onsets was measured to the nearest millisecond yielding the duration of the perceived interval.

**Figure 1.**
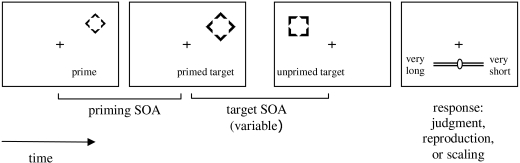
Succession of events in a sample trial of the experiments. The stimuli are not drawn to scale. Depicted is a scaling trial with the ruler in the lower part of the screen.

In the scaling part, a horizontal ruler appeared in the lower half of the display (see Figure 1). Its ends were labelled ”very long” and ”very short”. The position of the labels varied randomly from trial to trial. Participants moved a marker on the ruler with the mouse and adjusted the interval they had perceived. The ruler was 200 pixels long, and the relative position of the marker was measured. Participants were instructed to use the whole length of the slider and told that “very long” meant “among the longest intervals presented”.

Before each part of the experiment, the participants practised the respective task. In these 28 trials, no primes were used. Each target SOA was repeated twice in order to give the participants an occasion to learn the range of intervals. A trained student experimenter gave occasional feedback if he or she saw that the participants did not use the upper part of the scale in the scaling task or produced very large intervals in the reproduction task. I did not use formal feedback because preliminary experiments with the same me-thods had consistently shown that the participants found all three tasks natural and very easy, an impression confirmed by the data.

### Computation, parameters and statistical analysis

**Figure 2.**
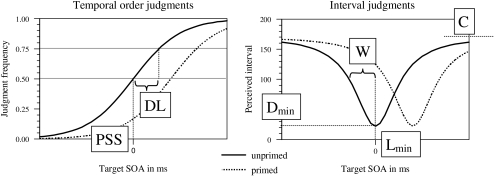
Distributions expected in the TOJ (left) and in the scaling and reproduction task (right). Solid lines depict data expected in unprimed trials, dotted lines depict data expected in primed trials. PLP is evident from a shift of the distribution towards the right. Parameters are indicated on the figures. For a more detailed description, see the text and Appendix A.

Binary psychophysical judgments are typically distributed as a cumulative normal or a logistic function which is defined by two parameters, the point of subjective simultaneity (*PSS*), and discrimination accuracy (*DL*; see Figure 2 for an illustration). The *PSS* is the location on the abscissa at which the two judgments are equally likely, that is, the observers cannot decide about the temporal order. *DL* is defined as the interquartile range. From the data, the frequency of the judgment “unprimed stimulus first” was calculated, and *PSS* and *DL* were computed by logit analysis (Finney, 1971). Further, median judgment times were calculated for each SOA and priming condition.

The scaling and reproduction data should yield U-shaped distributions (see Figure 2). For illustration, consider the unprimed trials. The minimum interval or duration should be perceived when the targets are simultaneous. From the value of the minimum, the perceived interval should increase monotonically with the actual interval to some maximum value. Such a distribution can be approximated by a rational, nonlinear function with 4 parameters (see Appendix A for mathematical details). Parameter *L_min_* gives the location of the minimum on the abscissa which is comparable to the *PSS*. Recall that the *PSS* is the point of perceived simultaneity, which should be identical with minimum duration between the two targets; *L_min_* is the point of minimum duration. PLP should thus show up in a shift of *L_min_*.

Parameter *D_min_* reflects the perceived duration of the minimum (y-value of the minimum). It has no equivalence to psychometric analysis since there, the y-value of the *PSS* is by definition 0.5. By contrast, *D_min_*, the minimum duration perceived in a set of conditions, is not confined to a certain value or range. Parameter *W* is defined as the width of the opening of the U and possibly closely related to discrimination performance, that is, *DL* in classical psychophysical analysis (see Appendix A for a mathematical argument). Parameter *C* denotes the y-value against which the two branches of the U converge. It also has no equivalent in psychometric analysis since there, it is assumed that the psychometric function converges towards 0 and 1. Median individual scaling and reproduction results were calculated (excluding reproduced intervals longer than 1,000 ms) and, minimising least squares, the best-fitting function was approximated with Mathematica 4.1 (2001).

*PSS* and *DL* from the TOJ part, and each of the four parameters of the reproduction and scaling part were submitted to t-tests. PLP values (computed as *PSS* differences between the primed and the unprimed condition for the TOJ part and as *L_min_* differences for the two other parts) were submitted to a one-way repeated-measures ANOVA with the factor task. Judgment times were submitted to a two-way repeated-measures ANOVA including target SOA and priming as factors. If appropriate, degrees of freedom were corrected by the Greenhouse-Geisser-coefficient ε, and adjusted α values are reported (Hays, 1988).

### Results

#### Experiment 1a: Priming interval of 67.5 ms

**Figure 3.**
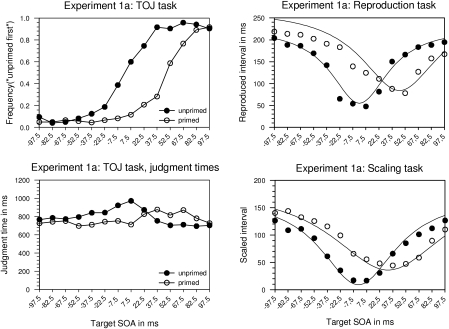
Results of Experiment 1a. Priming SOA is 67.5 ms. Lines in the graphs for the reproduction and scaling tasks represent the approximated function and were computed using averaged parameters of the subjects and the function described in Appendix A.

One participant always pressed the same button in the TOJ task; his data were not analysed. Figure 3 gives the mean data. There is an obvious shift of the primed distributions in all tasks. Table 1 details the statistical results which are summed up below.

**Table 1.** Statistical results of Experiment 1a and Experiment 1b. The first 6 rows give the t-tests of the parameters computed from the three tasks, the lower 2 rows the ANOVAs of the judgment times.

	*PSS* / *Lmin*	*DL* / *W*	*Dmin*	*C*
1a, TOJ	*t*(9) = 16.26, *p* < .0001	*t*(9) <1		
1a, reproduction	*t*(9) = 13.05, *p* < .0001	*t*(9) = 1.36, *p* = .21	*t*(9) = 1.2, *p* = .26	*t*(9) = 1.32, *p* = .22
1a, scaling	*t*(9) = 16.65, *p* < .0001	*t*(8) = 2.27, *p* = .053	*t*(9) = 4.61, *p* < .01	*t*(9) = 1.12, *p* = .29
1b, TOJ	*t*(10) = 7.12, *p* < .0001	*t*(10) < 1		
1b, reproduction	*t*(10) = 14.9, *p* < .0001	*t*(9) = 1.9, *p* = .09	*t*(10) = 1.72, *p* = .12	*t*(10) < 1
1b, scaling	*t*(10) = 11.41, *p* < .0001	*t*(9) = 2.05, *p* = .07	*t*(10) = 4.17, *p* < .01	*t*(10) < 1
	*Target-SOA*	*Priming*	*Interaction*	
1a, judgment times	*F*(13, 117) = 8.74, *p* < .001, *MSE*= 11407.3	*F*(1, 10) = 0.00, *MSE*= 3949.87	*F*(13, 117) = 2.76, *p* < .05, *MSE*= 4496.27	
1b, judgment times	*F*(13, 130) = 7.99, *p* < .01, *MSE*= 1246.7	*F*(1, 10) = 0.06, *MSE*= 5587.07		

##### TOJ task.

*PSS* were reliably shifted in favour of the primed stimulus (+48 ms compared with +1 ms in the unprimed condition). PLP thus was +47 ms. I did not find a significant influence of the prime on judgment accuracy; mean *DL* was 34 ms. As expected, the judgment times yielded a significant main effect of target SOA and an interaction of priming and target SOA. Judgment times peaked at +7.5 ms in the unprimed condition. The maximum in the primed condition was in the range of +37.5 to +67.5 ms, but shallow and double-peaked. The judgment times thus do not permit easy or unambiguous estimation of the location of the *PSS*.

##### Reproduction task.

As expected, the reproduced intervals yielded a U-shaped distribution (Figure 3). Also, a horizontal shift of the distribution in the primed condition is visible. Minimum location (*L_min_*) differed between the two conditions (0 ms in the unprimed and +46 ms in the primed condition). PLP thus was +46 ms. For *W* (average 47 ms), *D_min_* (average 70 ms), and *C* (average 249 ms), no significant influence of the prime was found.^2^ 2 In sum, a reliable influence of priming was found for the location of the minimum only.

##### Scaling task.

The statistical results were similar. *L_min_* was reliably influenced by priming (–2 vs. +32 ms) yielding PLP of +34 ms. *W* just failed to reach significance (average 66 ms). *D_min_* was reliably smaller in unprimed (9) than in primed trials (36). *C* was on average 181 ms and did not change with priming.^3^ In addition to PLP, the scaling task thus revealed a change in the perceived minimum duration.^4^

##### Comparison.

Individual PLP values for all three parts were submitted to a one-way repeated-measures ANOVA which reached significance, *F*(2, 18) = 15.73, *p* < .001, *MSE* = 37.42. Bonferroni post-hoc comparisons indicated that PLP was reliably smaller in the scaling task than in both other tasks, *p* < .05. Shore et al. suggested (2001) a method for computing true latency effects and response biases when comparing two measures which are influenced by a response bias to different degrees: PLP is the mean of the two conditions, the response bias is estimated as half the difference between the two conditions. Applying this method, the response bias in the present experiment could be estimated as 6.5 ms and the true latency gain as 40.5 ms, comparing the TOJ and the scaling task. Comparison of TOJ and reproduction yields a marginal response bias of 0.5 ms and a true PLP effect of 46.5 ms.

#### Experiment 1b: Priming interval of 37.5 ms

Experiment 1b was identical with Experiment 1a apart from that the priming SOA was reduced to 37.5 ms. This should also decrease PLP because attention as well as retouch has less time to operate. One participant was not able to discriminate order in the TOJ task (*DL* > 1,000 ms). His data were not analysed.

##### TOJ task.

*PSS* varied with priming. *PSS* were +7 and +39 ms, and PLP thus amounted to +32 ms. Mean *DL* was 24 ms. Judgment times yielded a significant main effect of target SOA and an interaction of priming and target SOA. They peaked shallowly at –7.5 to +7.5 ms in the unprimed condition, and at +22.5 to +37.5 ms in the primed conditions, again rendering an estimation of the *PSS* difficult.

##### Reproduction task.

*L_min_* was 0 ms in the unprimed and +26 ms in the primed conditions yielding a reliable difference. *W* just failed to reach significance (44 vs. 51 ms) which was also true for *D_min_* (26 vs. 35 ms). *C* did not vary with priming (average 213 ms). PLP thus was found, accompanied by a change in the duration of the minimum; a change in parameter *W* is indicated, but not established, by the present results.

##### Scaling task.

The statistical results were similar. *L_min_* was 0 ms for the unprimed, and +20 ms for the primed conditions yielding a significant difference. *W* again just failed to reach significance (42 vs. 62 ms). *D_min_* was influenced by priming and was 0 for the unprimed and 19 for the primed condition. *C* did not change with priming (average 167). This pattern closely resembles that of the reproduction part. PLP was again accompanied by changes in the perceived duration of the minimal interval.

##### Comparison.

Individual PLP values for all three parts were submitted to a one-way repeated-measures ANOVA which just failed to reach significance, *F*(2, 20) = 4.04, *p* = .06, *MSE* = 103.37. Using the proposal by Shore at al. (2001), we can estimate true PLP as 26/29 ms and response bias as 6/3 ms in the present experiment, for a comparison of TOJ with scaling, and reproduction, respectively.

##### Comparison across priming SOAs.

 Individual PLP values were submitted to a two-way ANOVA including priming SOA as a between-subjects factor and task as a within-subjects factor. Both were highly significant, priming SOA: *F*(1, 19) = 31.1, *p* < .0001, *MSE* = 129.9, task: *F*(2, 19) = 8.3, *p* < .001, *MSE* = 72.13. The interaction was also significant, *F*(2, 38) = 5.77, *p* < .01, *MSE* = 416.51.

## GENERAL DISCUSSION

The present experiments attempted to ascertain whether the possible facilitating influence of a masked prime on the temporal perception of the mask – predicted by two explanations of metacontrast masking and proven in numerous studies – is due to a response bias. New methods – the analysis of judgment times of the *TOJ*, *interval reproduction*, and *interval scaling* – allow the disposal of weaknesses in earlier attempts to address the response–bias question. I also aimed at providing phenomenal measures of the influence of a masked prime on the temporal features of the mask.

In the following, I will (1) summarise the evidence against the response–bias explanation of PLP from the present, and other, experiments, followed by (2) a closer look at the possible influence of priming on the parameters *D_min_* and *W* of the scaling and reproduction tasks. Finally, (3), I will return to the question as to how the influence of the masked prime on the temporal features of the mask can be integrated into models of masking.

### The role of response bias

PLP was found in all three tasks which were compared in the present study. This finding makes clear that PLP cannot be explained fully by a response bias, as, for instance, Pashler’s argument suggests (Pashler, 1998) by reason that such a response bias cannot operate in the reproduction and scaling tasks. However, it would be too far–reaching to conclude that response biases play no role at all in PLP. By contrast, there are some findings which might be interpreted as biases: In Experiment 1a, PLP was numerically (though not statistically) smaller in scaling than in the two other tasks; in the second experiment, the difference reached significance and also appeared (though not reliably) in the comparison of the reproduction and TOJ tasks. These differences might be the consequence of a bias which enlarges the effect of the prime in the TOJ task, but not in the other two tasks. At present, however, this is a tentative assumption because the difference was reliable only in one of two experiments for the scaling task and could not be proven statistically for the reproduction task.

On the other hand, besides this small and unreliable possible bias effect, the present experiments prove a genuine and large PLP effect. Earlier data support this conclusion (Scharlau, 2004). In that study I followed the logic of Shore et al. (2001) and changed the criterion between blocks, that is, I had the observers report which stimulus was the first one in one block and which was the second one in another block. I did not find a difference in PLP between blocks, that is, there was no bias, even numerically. This was in contrast to Shore et al., who reported a small criterion effect. I suggest that this bias was not found because masked primes afford less opportunity for decision-level influences as compared to visible cues, which were used by Shore and coworkers.^5^ If this interpretation could be corroborated, it would be a general argument in favour of the use of masked primes or cues.

### New effects of priming on temporal perception

The novel methods hinted at additional effects of a masked prime: increases of W, and of *D_min_*. *W* relates to the width of the opening of the U-shaped function, *D_min_* is the y-value assigned to the minimum of the function. The increase of *D_min_* is proven only for the scaling task, the enlargement of *W* was reliable only in the scaling task of Experiment 1b and failed to reach significance for the reproduction tasks. Might these data add to the explanation of PLP? I suggest that this question should be carefully considered.

**Figure 4.**
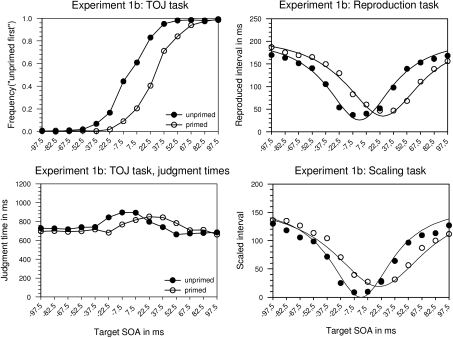
Results of Experiment 1b. Priming SOA is 37.5 ms. Lines in the graphs for the reproduction and scaling tasks represent the approximated function and were computed using averaged parameters of the subjects and the function described in Appendix A.

In the TOJ data of the present experiments, discrimination accuracy (*DL*) was the same in primed and unprimed trials. This is in accordance with earlier studies, in which a change of *DL* was a very rare exception (e.g. Scharlau, 2002, 2004; Scharlau & Neumann, 2003a, b). PLP assessed by the TOJ thus is confined to a *PSS* shift. If this also were true in the novel tasks, the only change between the unprimed and the primed distribution should be a horizontal displacement. By contrast, the distribution of reproduced and scaled intervals was seemingly flattened in primed trials (see Figures 3 and 4). (It may be noted in passing that this accords qualitatively with the judgment times, whose distribution was also less pronounced in primed compared to unprimed trials.)

This apparent flattening is reflected in two quantitative findings: Parameter *W* tends to increase in primed trials, that is, the U-opening is wider. An enlargement is also found for parameter *D_min_*. *C*, however, the axis of convergence, does not differ in primed and unprimed trials.

The small increase in *W* might indicate reduced discrimination accuracy. Note however, that the relationship of *W* to accuracy is not simple. A large *W* (a very broad opening) indeed indicates poor accuracy. By contrast, a small *W* (a very narrow opening) does not indicate excellent accuracy but rather a categorical use of the response alternatives: small perceived intervals within a small range of short SOAs and large, only slightly changing perceived intervals with other, more extreme SOAs. However, the numerical range of the *W* values found in the present experiments (which were neither very large nor very small) possibly indicates a decrease of accuracy, that is, the observers were less good in their duration judgments when a prime was present.

Since the decreased accuracy was only a trend, it should be interpreted cautiously. Future research might aim at corroborating this finding and investigate why it is absent in the classical TOJ task. For instance, it could be tested whether TOJ performance is so easy that the prime’s presence is not detrimental for temporal perception. One likely reason for this argument is that spatial clues are useful for TOJ. For instance, participants may have utilised apparent motion (Kolers, 1972) for their judgments of temporal order (Allik & Kreegipuu, 1998). Primes provide spatial information and thus might have fostered temporal judgments. The same spatial clues are less beneficial for the other tasks, which require estimating durations: The duration cannot (or, to be very cautious, can less easily) be inferred from apparent motion. That is, a possible detrimental influence of the prime, which may have impaired duration estimation, might have been compensated for by the spatial clues in the TOJ.

Let us now turn to the parameter *D_min_*. I interpreted it as the perceived duration of the minimum interval. In unprimed conditions, *D_min_* was close to zero in the scaling task, and only slightly larger in the reproduction task. *D_min_* was reliably increased in the primed trials of the scaling task, and numerically (though not statistically) enlarged in the reproduction task. Interpreted in perceptual terms: There was some minimal perceived interval, but its perceived duration was different from zero in the primed trials.^6^

This latter interpretation entails an interesting hypothesis: None of the durations perceived in the primed trials seems to be something like “subjective simultaneity”. Subjective simultaneity of the two targets should result in a perceived interval of zero. I suppose that the increase in *D_min_* indicates that simultaneity is only rarely registered in primed trials. There is always an additional onset, that of the prime, and some information about this extra onset may be available and foster the impression of non–simultaneity. This explanation accords well with earlier experiments which showed that observers use an additional judgment “simultaneous/unclear” less often in primed than unprimed sequences (Scharlau, 2004; Scharlau et al., 2006). By contrast, the TOJ task does not require the observers to process the duration of the perceived interval, and it is useless for the two-alternative forced-choice judgment to register simultaneity. This reading of the data dovetails with temporal perception models, which incorporate simultaneity or synchrony as a stand-alone category in addition to temporal orders, for example, Jaśkowski’s (1991) two-stage model of order perception.

It is important to note that two–alternative forced–choice judgments (as used in the TOJ task) are not apt to detect such changes in temporal perception because the *PSS* is by definition the target SOA at which the two judgments are equally likely. In addition to three–alternative forced–choice judgments (including a “simultaneous/unclear” alternative; Scharlau et al., 2006), the two methods tested in the present study provide a method which is sensitive enough to detect such changes.

If my reading of the *D_min_* data is correct, we should ask whether *L_min_* and possibly also *PSS* should be interpreted as the *point of subjective simultaneity* as classical psychophysical theory assumes (cf. Woodworth & Schlosberg, 1961). They might instead indicate a point of *maximal uncertainty*. The shallow peaks of the judgment-time distributions corroborate such an interpretation: The elevation of judgment times marks the interval of uncertainty, but not the *PSS*.

### PLP and models of masking

Let me now turn to the final question: How might the present data contribute to the understanding of masking? To sum the findings up in one sentence, a masked prime influenced the perceived temporal features of the mask. At present, two types of explanations can integrate this finding, an inheritance explanation and two–process models.

Inheritance denotes a process by which figural features of the prime are transferred to the mask (see, e.g., Werner, 1935, for some examples). Herzog and coworkers have demonstrated inheritance for a vernier offset of the prime (e.g., Herzog, Fahle, & Koch, 2001; Herzog, Koch, & Fahle, 2001). Such a mechanism might also operate in the temporal domain and transfer the perceived onset of the prime to an object–level representation of the mask. As yet, inheritance models do not include temporal information as an explicitly coded feature, but they also do not preclude it. Besides the present data, at least one further study has found inheritance of subthreshold temporal information (Elliott, Shi, & Sürer, in press).

Perceived time could also be part of object–level rep-resentations in reentrant processing, which has been suggested as an explanation of masking, for example by Di Lollo, Enns, and Rensink (2000). Latency facilitation of the mask suggests that the prime’s temporal information survives reentrant overwriting of the content of these object–level representations. It might be noted in passing that this hypothesis would resolve an ambiguity in the object–substitution (Di Lollo et al., 2000) or object–updating (Enns, 2002; Moore & Enns, 2004) account: Some authors suggest that detection of inconsistencies between the higher-level interpretation initiated by the prime and the later input of the mask causes abolishment of the initial ‘object token’ of the prime (Jiang & Chun, 2001), whereas others imply that object files are updated rather than created anew (Lleras & Moore, 2003). PLP might be interpreted as showing that temporal information of the prime persists throughout reentrant updating. Thus, the hypothesis of Lleras and Moore fits better with the present evidence.

The explanations from feature inheritance and from reentrant processing are not mutually exclusive. Far from it: Feature inheritance and related phenomena such as masking might be by–products of reentrant processing (Hamker, 2006). Hamker (this volume) has further shown that feedback loops may be sufficient preconditions for finding orientation inheritance. More importantly, feature inheritance can be explained by a model which was originally designed for a different purpose, namely explaining feature-based attention and goal-directed visual search. Whether this also holds for temporal inheritance is yet unclear.

Second, two–process models of the emergence of stable percepts in the processing of fast spatio-temporal input sequences might explain the findings via the interaction of a fast feature coding process and a slower consciousness–related upgrading process. Two of these models, *asynchronous updating* (Scharlau & Neumann, 2003a) and *perceptual retouch* (Bachmann, 1994), have already been described in the Introduction. In addition, *object substitution or updating* might also be regarded as a two–process explanation (Enns, 2004). Assuming that the perceptual history of an object begins with its *entry* into the reentrant process (not with the success of reentrant object formation), reentrant accounts could explain PLP; early visual coding would thus be the first process, reentrant hypothesis testing the second, higher process.

What is more, PLP might be used to infer the duration of reentrant processes. If the interval between prime and mask is long enough to terminate establishment of the prime’s object file, no PLP should be expected for the mask. Studies varying priming SOA and establishing the time course of PLP can provide such evidence (Scharlau et al. 2006; Scharlau & Neumann, 2003b). It should be noted in passing, however, that the object–updating account might at present not be apt to explain why PLP is the same for masked as well as non-masked primes: Non–masked primes should cause an object file of their own, separate from the mask’s object file, and in contrast to masked primes.

None of the two–process models is very specific about the processing of temporal information. The notion of two–process models suggests that the point in time at which higher–level processing of a stimulus (i.e., attention or upgrading) starts is equivalent to the perceived onset of the stimulus. If this hypothesis holds, the present experiments can be interpreted to indicate that this equivalency also holds for the interval between the onset of processing of the first and the onset of processing of the second stimulus on the one hand and the perceived interval on the other hand. However, the construct ‘time of perception’ is not necessarily equivalent to the perception of time (for a general discussion, see Neumann & Niepel, 2004). In fact, the AUM even argues that time of perception cannot be operationalised by perception of time (Neumann, 1982). My above argument that PLP never leads to perceived simultaneity between asynchronous targets, not even if the amount of PLP exactly compensates for the asynchrony, might support this conclusion.

While two–process models are not sufficiently fleshed out to address this problem, inheritance explanations (including the above interpretation of reentrant processing) do not suffer from a like ambiguity because they exclude the topic of time of processing and exclusively address the topic of perceived time. In terms of a general theory of temporal perception, they are thus limited, as compared to two–process models, although both types of model can equally well explain the present findings.

Let me conclude with some remarks on phenomenology. Studying the phenomenology of the masked as well as the masking stimulus was a prominent and natural method in early masking research (e.g., Werner, 1935). Observers reported what the prime (or mask) looked like. Only later was this method abandoned for the benefit of forced–choice detection or discrimination which, for instance, allowed discriminating between sensitivity and bias of the observer. These days, phenomenology sounds like a difficult, possibly philosophical and 19^th^ century enterprise. Yet, this is not true. The present study aimed at showing that it is easy to assess the phenomenology of masking and that one can do this entirely within the realm of classical psychophysics. If the results are reliable, a masked prime changes the perceived onset of the mask on a quite general level, and it induces a non–simultaneous component into the perception of the mask and the standard stimulus even if the two are simultaneous. This is, of course, not a very rich phenomenology, but it is a first step towards a reorientation of masking research on perception.

## Notes

^1^In the study of Shore et al. (2001), latency facilitation is termed “prior entry”, in accordance with a long–standing research tradition on attentional facilitation on the time of conscious perception (Wundt, 1887; see also Titchener, 1908). I do not use the term “prior entry” because it often refers to bimodal TOJ and attention and rarely to control of attention by cues, and because it has not been related to the processing of non-conscious information. Also, “prior entry” would suggest an attentional explanation of the phenomenon which is very likely but not established beyond discussion (Scharlau, in press).

^2^One participant’s approximation yielded a *C* value of more than 10,000 ms, which was omitted, see also the two *W* analyses in Experiment 1b.

^3^Note that in the scaling task *D_min_* and *C* are defined by the scale (0–200), and not in ms.

^4^It is possible to extend the regression of the psychometric functions to additional parameters, for example, by including the two axes of convergence into the regression and computing the *PSS* at the *p* value that lies exactly in between them. However, the psychometric distributions of the present experiments show that this is not necessary. In Experiment 1b, the functions doubtless converge to 0 and 1. In Experiment 1a, the convergence is slightly less clear, but still evident.

^5^Pashler (1998) pointed out that much of the evidence of attentional facilitation may be due to an inhibitory mechanism which allows disregarding irrelevant information, that is, *noise-reduction*. This latter account may explain the results of the TOJ task, because here it suffices to attend to the first stimulus and report its shape. However, it cannot as easily be applied to the other tasks, because estimating the interval requires comparing the onsets of *both* targets. It is thus impossible to block or disregard information from the unprimed channel. Thus, noise-reduction cannot possibly account for PLP.

^6^In order to corroborate this interpretation, I tested the *D_min_* values against zero in the two scaling tasks. In both experiments, no difference was found in the unprimed trials (both *t*s < 1). In primed trials, *D_min_* was larger than zero (both *t*s ≥ 3.41, both *p*s < .01). In the reproduction part, all *D_min_* values differed from zero (all *t*s ≥ 2.93, all *p*s < .05), in all likelihood because of difficulties in pressing the two mouse buttons at exactly the same time.

## Appendix A

I used the nonlinear, rational function

f(x) = a + b ×(1 - (1/(1+ c² × (x - d)²)))

and a least square regression for computing the parameters which are defined as follows.

*L_min_* = d (location of the minimum on the abscissa)

*W* = 1 / (width of opening)

*D_min_* = a (y-value of the minimum)

*C* = b + a (axis of convergence)

In this function, *W* is defined by the point of inflection on each branch of the U (the distance between the point of inflection and the location of the minimum). In logit analysis, *DL* gives the mean slope in the inner quartiles of the distribution, that is, between 0.5 and 0.75. The point of inflection on the logistic curve is only marginally different from *DL* (0.78). *W* thus is numerically similar to *DL*.

Note that we used this function because of its prima facie fit with the data, not because of a theoretical reason (as with the logistic function for the TOJ data). We first tested a reversed normal function with two additional parameters (*C* and *D_min_*) which was not as good as the present one, but of course, empirical supremacy requires that more functions than two have been compared.

Thus, I cannot and indeed do not want to claim that this function is the best one for approximating scaling and reproduction data. It yields reasonable results, especially for the parameter of main interest, *L_min_*, and the overall quality of fit is acceptable (see Figures 3 and 4, right columns). This suffices for the aims of the present study, and the function should thus be regarded as a candidate for future validation.
